# Adapting and Evaluating a Theory-Driven, Non-Pharmacological Intervention to Self-Manage Pain

**DOI:** 10.3390/healthcare12100969

**Published:** 2024-05-08

**Authors:** Jennifer Kawi, Chao Hsing Yeh, Lauren Grant, Johannes Thrul, Hulin Wu, Paul J. Christo, Lorraine S. Evangelista

**Affiliations:** 1 Cizik School of Nursing, University of Texas Health Science Center at Houston, Houston, TX 77030, USA; 2 Kirk Kerkorian School of Medicine, University of Nevada, Las Vegas, Las Vegas, NV 89106, USA; grantl1@unlv.nevada.edu; 3 School of Public Health, Johns Hopkins Bloomberg, Baltimore, MD 21205, USA; jthrul@jhu.edu; 4 School of Public Health, University of Texas Health Science Center at Houston, Houston, TX 77030, USA; hulin.wu@uth.tmc.edu; 5 School of Medicine, Johns Hopkins University, Baltimore, MD 21205, USA; pchristo@jhmi.edu; 6 School of Nursing, University of Nevada, Las Vegas, Las Vegas, NV 89154, USA

**Keywords:** theory-driven intervention, chronic pain, self-management, auricular point acupressure, non-pharmacological intervention

## Abstract

Background: The existing literature has limited detail on theory-driven interventions, particularly in pain studies. We adapted Bandura’s self-efficacy framework toward a theory-driven, non-pharmacological intervention using auricular point acupressure (APA) and evaluated participants’ perceptions of this intervention on their pain self-management. APA is a non-invasive modality based on auricular acupuncture principles. Methods: We mapped our study intervention components according to Bandura’s key sources of self-efficacy (performance accomplishments, vicarious experience, verbal persuasion, and emotional arousal) to facilitate the self-management of pain. Through a qualitative study design, we conducted virtual interviews at one and three months after a 4-week APA intervention among 23 participants using purposive sampling to describe their experiences in managing their pain based on our theory-driven APA intervention. Results: Using thematic analyses, we found four themes: the enhanced self-management of pain, improved pain outcomes, the feasibility of technology, and the sustainability of APA. Conclusions: Describing how interventions are mapped according to the elements of theoretical frameworks can help to guide intervention development, advance science and knowledge development, and promote the implementation of interventions. As such, using Bandura’s self-efficacy theory as a foundation for the APA intervention, APA was found to be feasible and sustainable, improving self-management, pain intensity, and pain-related outcomes. Participants provided recommendations for the further improvement of this theory-driven intervention.

## 1. Background

Although multiple pharmacologic and non-pharmacologic interventions are available, the high prevalence of chronic pain continues to persist [1], affecting more than one in five adults in the United States and worldwide [2,3]. One way to address the challenges in current pain management is to frame interventions within historically evidence-supported theories to drive elements of the intervention toward greater potential for success [4]. Unfortunately, with a limited focus on theoretical frameworks, many studies do not provide detailed information on how their interventions were founded on theory [4].

We adapted and applied Bandura’s self-efficacy theory [5,6] to a non-pharmacological intervention (auricular point acupressure (APA)) as a possible solution for the effective self-management of pain. Auricular point acupressure is acupuncture-like, needleless stimulation that uses pellets (0.2 × 0.2 mm size, non-medicinal Vaccaria seeds) embedded in tape and applied to ear points, which are pressed by the user at least three times per day for 3 min each time (9 min total per day); there is long-standing support for APA’s impact on pain relief [7,8]. In order to increase APA’s accessibility, we designed, developed, and pilot-tested a smartphone app as a self-guided tool for the self-administration of APA as a chronic pain self-management modality [8]. We chose Bandura’s self-efficacy theory because in order to self-administer APA, individuals needed to know how to locate their active ear points based on standardized recommendations and systematic APA techniques and then apply the seeds to the identified ear points to achieve a therapeutic effect [9]. Self-administered APA to manage pain can be influenced by one’s self-efficacy. Self-efficacy, defined as an individual’s confidence or belief in the ability to perform activities that are necessary to achieve an end goal, is an important prerequisite for self-management. Self-efficacy is a key variable that helps to explain the mechanism by which self-management (i.e., APA practice) occurs, hence mediating pain outcomes [10,11,12]. The Institute of Medicine strongly recommends self-management [1,13], which is described as the execution of tasks and skills through self-efficacy to motivate and encourage patients to make appropriate decisions and engage in health-directed behaviors [14]. 

Therefore, the aims of this paper were to discuss (1) how we adapted and applied Bandura’s self-efficacy theory in our APA intervention and (2) how the participants described their experiences in using our theory-driven APA intervention for their pain. These aims are important because the articulation and application of theory can help to advance knowledge development and guide interventions. This is especially significant in chronic pain, which continues to negatively impact our societies globally.

Chronic pain refers to an unpleasant experience occurring for at least 3 months or longer, beyond the typical healing time [1]. Pain that is chronic becomes more problematic to manage adequately. For example, more than one in three adults with chronic pain develop “high-impact” chronic pain, suffering from frequent limitations in their daily lives and work productivity [2]. Consequently, the total direct and indirect healthcare costs are extremely high, surpassing the costs of managing other major chronic health conditions, thereby creating an economic and public healthcare burden [15].

Despite the increased sophistication and technology in pain management, current treatments have found limited success. Systematic reviews of randomized controlled trials on non-pharmacologic and non-opioid pharmacologic modalities have concluded that there is only sparse evidence for interventions’ efficacy, most effect sizes are small, the interventions are of a short duration, and existing trials have small sample sizes [16,17]. Similarly, opioid medications for the management of chronic pain have shown small improvements in the short term, lack evidence for long-term use, and come with increased risks of harm, even when used briefly (one to less than six months) [18]. Taken together, these findings demonstrate that chronic pain persists as the leading cause of disability, and effective management continues to be a huge challenge [15].

The Institute of Medicine has long recommended self-management as a key strategy in addressing chronic pain, particularly since a complete cure is unlikely [1]. Self-management is necessary for the day-to-day care required in the lives of those affected by chronic pain. Patients with chronic pain who actively engage in their treatments achieve better outcomes than those who take a passive approach [19]. Given the complexity of behavior change interventions, theory-based behavioral interventions produce longer-lasting effects than those lacking a theoretical basis [20]. Hence, theoretically driven self-management programs are ideal and most recommended [21]. However, many published healthcare studies provide limited information on whether and how theories were applied in conducting their research, resulting in challenges in advancing knowledge development and in the ability to guide future practice [4]. Theoretical frameworks are valuable to guide the conductance of research, especially in intervention studies [4,22].

There are some theories specific to the area of chronic pain. For example, Dunn (2004) proposed a mid-range theory (mid-range theories are narrower in scope compared to broad, grand theories) based on Roy’s adaptation model, utilizing the process of theoretical substruction. Dunn identified concepts, assumptions, and relational statements from Roy’s model and formed the mid-range theory, Adaptation to Chronic Pain, which focuses on coping processes and adaptive modes toward pain control [23]. Lefort (2004) tested Braden’s Self-Help Model through an educational intervention (Chronic Pain Self-Management Program) to help individuals to manage their own pain and found favorable outcomes (e.g., pain intensity, self-efficacy) [24]. Orem’s Self-Care Deficit, Pender’s Health Promotion, and the Chronic Care Model have also been applied to chronic pain, among other conditions [25,26]. Some authors utilized theories to inform their pain self-management interventions [27,28,29]. However, details on how these were mapped within the intervention were mostly lacking.

Among existing theories that can be applied for pain management, we deemed Bandura’s self-efficacy to be most appropriate for our active, non-pharmacological intervention to self-manage pain utilizing APA. Self-efficacy, the cornerstone of effective disease self-management interventions [30], is a key variable that helps to explain the mechanism by which self-management functions as a mediator of treatment outcomes [10,11,12]. Bandura theorized that self-efficacy helps to determine whether behavior or actions can change or whether outcomes can be achieved by an intervention. Self-efficacy during a course of treatment helps to predict whether behavior change happens successfully. Therefore, in our studies [8,31], we adapted Bandura’s self-efficacy theory to guide a non-pharmacologic self-management intervention to address chronic pain using APA.

Auricular point acupressure (APA) is derived from the principles of acupuncture, which is now covered by Medicare and other insurers, with supporting evidence that it can be used to manage pain effectively [16]. However, acupuncture is difficult to scale up as it needs to be administered by acupuncturists, limiting its widespread implementation. On the other hand, APA, which is based on similar auricular (ear) acupuncture points, uses small seeds (instead of needles) secured into designated ear points to stimulate ear zones, allowing for easier access and the capacity for self-administration with adequate training. The ear zones represent specific body parts where individuals experience pain [32,33]. The underlying mechanism of APA suggests that the auricular nervous system is a microsystem that represents and mimics the entire body in such a way that specific ear zones have reflex connections with specific parts of the body. These assumptions have been supported using functional magnetic resonance imaging [34,35]. Our previous studies that included quantitative sensory testing [36], functional magnetic resonance imaging [37], and cytokine biomarkers [36] also help to explain the mechanisms by which APA successfully relieves pain.

In order to widely disseminate this promising APA intervention to self-manage pain, we have been using digital healthcare approaches. Digital healthcare is especially useful in times when there are limited opportunities for face-to-face interactions, in-person visits are minimal, or healthcare is simply inaccessible [38]. Digital healthcare refers to the broad application of various technologies, including health information technology, telehealth, mobile health, wearable devices, and personalized medicine [39]. Building upon the work of Bandura, we adapted and applied the self-efficacy theory to self-manage pain through APA using digital healthcare, as a possible solution in advancing the science and practice of pain management. This paper will detail how we adapted and applied Bandura’s self-efficacy theory in our APA intervention. Secondarily, we evaluate our theory-driven APA intervention using our overarching research question directed to the study participants: *“How were your experiences in using APA for your pain?”*

## 2. Methods

The current study was part of a longitudinal, waitlist, randomized controlled trial utilizing our theory-based APA intervention [40]. It was a multi-site trial conducted at universities and healthcare settings with diverse populations located on the East (Baltimore, Maryland, and surrounding areas) and West Coasts (Las Vegas, Nevada, and surrounding areas). Due to the large amounts of data, we report our quantitative results separately. The trial was registered on ClinicalTrials.gov (identifier: NCT05020470). Single Institutional Review Board approval was received (IRB00290512) and written informed consent was obtained prior to data collection.

The current study had a qualitative, descriptive design, chosen because it was aligned with the aims of this work and the need to present clear, direct, and easily understandable descriptions of participants’ experiences with APA for relevance and utility in intervention development and testing [41,42]. This paper was written based on the Consolidated Criteria for Reporting Qualitative Research [43].

### 2.1. Participants, Procedures, and Data Collection

In the trial from which this qualitative study was derived, participants were randomized into intervention (APA) and control groups (pain education). Participants in this qualitative study were purposefully selected (all belonging to the intervention group, based on the key aim of this work, which was to understand their experiences with APA). Participants were eligible if they (1) were 18 years or older, (2) were able to read and write in English, (3) had chronic pain for at least 3 months, (4) reported an average intensity of pain ≥ 4 on an 11-point numerical pain scale for the previous week, (5) were smartphone users, and (6) were able to apply pressure to the seeds taped on their ears. Participants were excluded from the study if they had any allergy to latex (due to the tape used to secure the seeds to the ear points).

Using study advertisements, recruitment occurred from November 2021 to January 2022. Through purposive sampling, we screened 30 participants for eligibility to participate in our study. Seven participants were excluded from the study (3 did not meet the inclusion criteria, 3 changed their minds, and 1 could not commit to the study duration of a 4-week APA intervention and 3 months of follow-up). Among the 23 remaining participants, 1 dropped out due to a busy work schedule, resulting in a 96% retention rate.

We conducted 1-month and 3-month post-intervention, semi-structured, individual, virtual interviews (approximately 30–45 min, with audio–video recording), having participants reflect on their experiences with our theory-driven intervention. The overall, open-ended question asked participants to describe their experiences in using APA for their pain. The interview guide included the following queries: (1) likes and dislikes related to APA, (2) its helpfulness or lack thereof, (3) any changes in the use of pain medications or healthcare utilization, (4) their experience with the app (likes and dislikes or any difficulties, suggestions for improvement), and (5) any other comments or recommendations regarding our theory-based APA intervention. The interviewers (JK, CHY, LG) were trained in conducting qualitative research; 2 were PhD-prepared professors and 1 was a graduate research assistant. The interviews were conducted remotely in a private setting based on the participant’s choice (e.g., home).

### 2.2. Intervention Mapping according to Bandura’s Self-Efficacy Theory

Our APA intervention was designed based on Bandura’s self-efficacy theory, which posits that self-efficacy helps to determine whether behaviors and activities are initiated and sustained to produce significant outcomes [6]. Accordingly, self-efficacy is derived from four key sources of information: *performance accomplishments*, *vicarious experience*, *verbal persuasion*, and *emotional arousal* [6]. The following sections provide comprehensive details regarding how we adapted and applied Bandura’s theory to develop our APA intervention’s components.

**Performance Accomplishments.** Performance accomplishments are based on the mastery of personal experiences [6]. With repeated success, self-efficacy is developed. On the other hand, failures can be overcome with determination, self-motivation, persistence, and sustained effort, toward developing self-efficacy. Performance accomplishments can be achieved through self-instructed performance, participant modeling (guided performance), performance exposure (repeated experiences), or performance desensitization (exposure to adverse experiences). We adapted performance accomplishments in our intervention through our smartphone application (“app”), with self-guided instructions to learn about APA. These self-guided instructions aimed to increase the knowledge and APA skills among participants and support them in developing their confidence toward the self-management of chronic pain. Using virtual telehealth-like sessions, we conducted participant orientation and training on APA, delivered by experienced research team members using a manualized and standardized intervention protocol, while allowing individualization depending on participants’ chronic pain needs. For example, some participants may have needed more time and repeat virtual sessions to facilitate the mastery of APA.

**Vicarious Experience.** The next source of information to facilitate self-efficacy is vicarious experience. The ability to see how others perform in tasks without adverse outcomes and with good results suggests that one might also improve if one develops the knowledge, performs the skill, and sustains such learned skill [6]. Vicarious experiences can be induced through modeling. For example, in our APA intervention protocol, we included a variety of short videos in the app, showing how to find appropriate ear points for specific pain areas, how to apply and secure the seeds to specific ear points, and how to press the seeds onto the ear points to create acupressure-like stimuli to facilitate pain relief. During the virtual training sessions, our research team members also modeled APA. Further, we shared participants’ successful experiences with others through weekly and monthly communications to help to motivate and sustain their APA use.

**Verbal Persuasion.** The third key basis of information to enhance self-efficacy is verbal persuasion. While verbal persuasion is not as impactful in enhancing confidence as personal accomplishments [6], individuals can develop greater self-efficacy when encouraged through suggestions, exhortations, repeated instructions, and corrective feedback. In our study, after the participants learned APA through the app with an initial virtual meeting for orientation and training, we advised them to start using APA. We asked our participants to take a photo of their ears after 2–3 days of APA use, showing the seeds in their ear points, based on the location of their pain in the body. We followed this with a repeat virtual session to provide encouragement, support, and revision of the ear point placement when necessary. Participants were also given tailored feedback messages through our app depending on their pain levels and APA use. Through brief pain surveys incorporated into the app, we were able to gather their pain levels, as well as the frequency and duration of APA use. Based on this information, we tailored data-driven motivational messages to facilitate continued and sustained APA use. Further, we facilitated digital literacy and confidence in the use of our app through return demonstrations and feedback [44].

**Emotional Arousal.** The last source of information from which self-efficacy is derived is emotional arousal. Individuals experience stress and anxiety in threatening situations, which impacts their self-efficacy, resulting in suboptimal performance, fear, and failure [6]. On the other hand, modifying self-limiting emotions through attribution, relaxation, biofeedback, or cognitive processing can reduce self-doubt, improve performance, and increase self-confidence. In our studies, we used virtual meetings to provide opportunities to answer participant questions and deliver self-management support by trained research team members. We also employed ecological momentary assessment (EMA) [8,31] to provide immediate feedback from the research team to the study participants. EMA is a method used to collect real-time data, as participants go about their daily activities, through short surveys delivered via smartphones. We employed EMA in our prior studies to allow for the real-time assessment of participants’ pain levels and their APA usage. Participants were instructed to click on a link that was texted to them at four random times during the day to evaluate their pain levels and APA use. These data were processed and visually displayed as graphic images in each participant’s app, allowing the participants to see and monitor their pain levels and APA performance data. According to Bandura [5,6], when individuals see their efforts and successes, these motivate them toward the further mastery of tasks, producing a greater sense of accomplishment and positive emotional arousal for enhanced competence. We then advised participants to continue their APA use for 4 weeks. We anticipated that some participants might struggle and experience setbacks in their APA use. Our EMA data allowed the research team to monitor participants’ progress and facilitate dynamic and immediate feedback. Further, our virtual meetings helped to assist and encourage behavior and actions toward independent performance to produce successful experiences that helped to reinforce their competency in APA.

**Figure 1:**
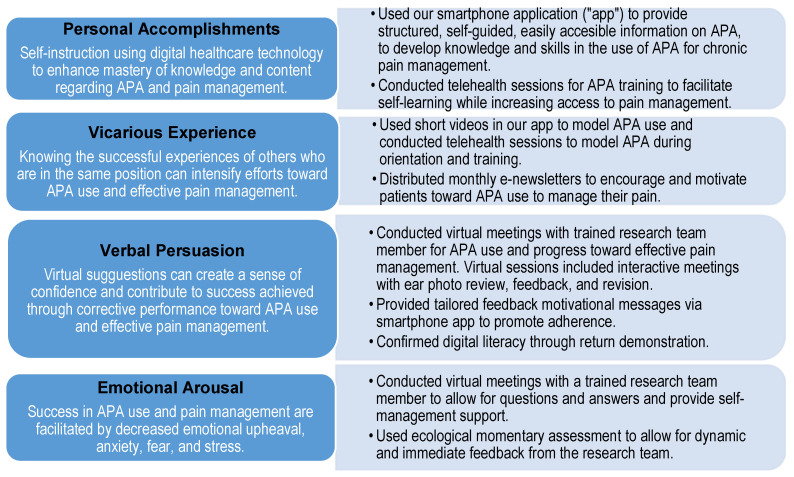
Theory-driven APA intervention based on Bandura’s self-efficacy sources.

In summary, Bandura’s self-efficacy framework provided a theory-based solution to inform APA delivered via digital healthcare technology to facilitate the self-management of pain. Since self-efficacy is necessary for self-management, Bandura’s four major sources (personal accomplishment, vicarious experience, verbal persuasion, and emotional arousal) by which one develops self-efficacy should be targeted in interventions to facilitate the successful self-management of pain. Bandura’s self-efficacy theory served as a blueprint to guide our APA intervention (see Figure 1).

The figure details Bandura’s self-efficacy sources (first column); each one is then addressed via our intervention (second column). With our comprehensive, theory-based APA intervention, participants can be empowered to successfully self-manage their chronic pain toward favorable outcomes. We expect that this 4-week theory-driven, comprehensive APA intervention will result in a reduction in chronic pain in clinical outcomes and psychosocial consequences consistent with the biopsychosocial model of pain [45]. Since a “cure” for chronic pain is unrealistic at this time, healthcare providers and patients rely on the self-management of pain [1]. Self-management is described and operationalized through three essential tasks [10], which are the putative targets for our studies, reflecting clinical and psychosocial pain outcomes: (a) *medical management* or the work needed to care for one’s chronic pain (e.g., measured through analgesic use, healthcare utilization); (b) *role management* or the work required to maintain quality of life despite the pain (e.g., measures of quality of life, self-efficacy); and (c) *emotional management* or the work needed to deal with one’s feelings (e.g., psychological measures).

### 2.3. Data Analysis

We used thematic analyses to identify recurrent themes based on the data derived [46]. Thematic and content analyses are commonly used in qualitative descriptive research, but the former was chosen for this study in order to provide a rich and detailed account of participants’ experiences with APA, with less emphasis on quantifying the qualitative data [42]. We followed Braun and Clarke’s systematic guidelines and steps for thematic analysis [46].

With NVivo, data collection and analyses were performed iteratively and simultaneously until data saturation was achieved. Recorded and transcribed data were independently reviewed and analyzed (JK, CHY, LG) through the initial thematic analysis steps: the repeated review of and familiarity with the data, the generation of codes, and searching for themes. Similar responses were labeled and coded based on participants’ replies to the interview guide (e.g., likes/dislikes, helpful/not). Codes were collated to develop themes as derived from the data. Next, all three researchers (JK, CHY, LG) communicated and convened several times to apply the subsequent steps of the thematic analysis: they reviewed the themes, described and identified the named themes, and prepared the report. In these last three steps, a recursive process was conducted to arrive at a consensus resulting in the emerging themes [46]. We used Braun and Clarke’s 15-point checklist in evaluating the quality of our analysis [47]. Further, to facilitate rigor [48], we performed the following: interviews were consistently conducted using our interview guide; following each interview, data were summarized and confirmed with the participants, with member checking during the 3-month interview, where data saturation was reached and no new themes were derived, providing justification for the adequacy of the sample size; researcher reflexivity, written notes, and audit trails were implemented; and a qualitative methodological expert was consulted to facilitate trustworthiness and ensure the integrity of the data.

## 3. Results

The mean age of our participants was 43; 78% (*n =* 18) identified as females, while the rest were males. There were 52% (*n =* 12) who were White, 13% who were Asian (*n* = 3), 13% who were Black (*n* = 3), and 22% (*n* = 5) were either American Indian/Alaska Native or Native Hawaiian/Pacific Islanders. Finally, 17% (*n* = 4) were Hispanic.

We found commonalities among the 22 participants, which showed the following themes: the enhanced self-management of pain, improved pain outcomes, the feasibility of technology, and the sustainability of APA (adherence and participant retention). These themes were confirmed during our 3-month interviews and no additional, relevant data were gathered.

### 3.1. Theme 1: Improved Self-Management of Pain

Our theory-based APA intervention seemed to have facilitated the *improved self-management of pain*. Participants reported feeling grateful for the opportunity to have an effective pain modality in their tool kit that was accessible to use anytime that they experienced pain. They expressed enjoying the opportunity to treat their pain with a tool that was within reach at home or work, wherever they were, by themselves, and did not involve pharmacologic remedies. They were able to take an active part in the management of their pain.

*I was able to take control of my pain, my pain level decreased so I was able to continue my activities. I liked being able to take my care into my own hands and have something specific I could do when my pain was flaring. I realized how much my whole life was set around my pain so why not do this for myself. Being able to act on my pain, I can then work, do my chores, exercise, and feel better about myself*

(Participant #11).

*Access to Traditional Chinese Medicine is great. It [APA] can be done anytime, especially in today’s culture where there are short attention spans and we need relief now. It was easy to use APA, and it’s very affordable*

(Participant #8).

*As my pain was going down from 4–5 to 2 [out of 10], I realized I did not have to use my pain medication like before and I am moving more and living. I talked to my pain doc about it and he was happy. He said continue on [with APA]*

(Participant #6).

*I decided to participate in a 5 K Turkey Trot and had a few issues with my knee but not my back. I am feeling good so far! I felt confident doing this walk, I would have been afraid before, but I paced myself well and finished it!*

(Participant #5).

*I did not know much about it [APA] but I wanted to learn more. Other than the app, I googled it and watched some videos in YouTube. I also talked to my PCP [primary care provider] but he said he does not know much about it but that it seems ok*

(Participant #12).

### 3.2. Theme 2: Improved Pain Outcomes

In describing their experiences using our APA intervention, participants reported having improved pain and pain-related outcomes. These included a decreased pain intensity resulting in the reduced use of pain medications, better sleep, and less anxiety. The reduction in pain intensity was consistent with the quantitative findings, with a mean decrease from 5.45 out of 10 (baseline) to 2.7 (sustained up to the 3-month follow-up).

*[I am] happy that it really helps relieve pain and helped for anxiety as well. [I] liked using APA, seemed to get more relief with tightness and insomnia, definitely felt much more relaxed and sleeping better especially which is a big deal. APA was helpful especially with pain flare-ups*

(Participant #5).

*I found APA very helpful in that my pain level decreased considerably. I liked APA overall because it is easy, I did not have to go anywhere, [it was] user-friendly, accessible, I did not have to take meds. And yes! Quite a bit of decreased visits to my provider, I appreciated the savings from co-payments as well*

(Participant #6).

### 3.3. Theme 3: Feasibility of Technology

For this theme, we found supporting evidence in key components of our theory-based intervention. We found that our intervention using digital healthcare technology was helpful, easy, and practical. However, some participants were challenged in accurately locating the ear points for seed placement. Repeat viewing of the APA information and videos in the app, as well as the virtual sessions with ear photos, seemed to assist them significantly. In addition, although some appreciated the tailored motivational messages and self-monitoring of their pain level and frequency/duration of APA use, there were recommendations suggested for improvement.

*The app was great, [it] had all the information. I liked the simplicity of the intervention once the seeds were on and the videos were helpful. It was easy to use*

(Participant #14).

*The app was very helpful, useful, [I] watched several times especially to help with [seed] placement. I liked having direct contact with an expert in acupressure and I thought that was important because the ear seed placements can be tricky. I liked the structured, follow-up texts [EMA] and emails, virtual sessions, and monthly contacts since they helped me to stay on track and they did hold me accountable as a reminder to practice APA*

(Participant #4).

*Having me go through the app and try APA was good so I know I am doing it right. The motivational messages seemed to help, any new habit is hard so any encouragement would be helpful to continue to do it [APA]. They weren’t fancy though, maybe quotes from known people would be more inspiring. The graphs were helpful so I can see my pain level throughout but they were small on a phone, maybe a computer would be better*

(Participant #15).

### 3.4. Theme 4: Sustainability of APA (Adherence and Participant Retention)

We also found that *APA can be sustainable*, particularly from the results of our 3-month interviews focused on APA adherence and participant retention. We also noted some challenges that our participants experienced as they underwent our intervention. These included ear sensitivity from the seeds, hair becoming stuck in the taped seeds, or feeling self-conscious about the seeds in their ear. Some needed initial help in applying the seeds to the appropriate ear points or found the seeds to be slightly bothersome when lying down, which gradually improved. However, participants stated these to be minor inconveniences that they overcame, especially given the pain relief that they experienced overall.

*I really was impressed with the treatment as it really helped in decreasing my sciatic pain till the end of the study. I like that it seemed to help alleviate my back pain, [I am] able to do it consistently and conveniently on my own time with one application lasted so many days. Treatment allows for individualization based on pain area and sensitive spots in ears, [it is] simple, straightforward and I liked that it wasn’t a huge time commitment, sticking with it to the end*

(Participant #20).

*It was a little hard for me to find the spots as someone who had never done it on myself before. Getting the seeds in place was a bit awkward at first but after a couple of times, I found that I was able to do it quite easily and didn’t have to put as many on. Once I got used to it, it became easier after a while and [I] could tell difference in pain*

(Participant #1).

*I know that I will continue to use this even after this study because I have no insurance and I have very little income*

(Participant #18).

## 4. Discussion

We adapted Bandura’s theory, applying his four vital sources of self-efficacy (personal accomplishment, vicarious experience, verbal persuasion, and emotional arousal), and operationalized each of these in our APA intervention (see Figure 1). Our intervention components, mapped based on Bandura’s self-efficacy theory, seemed to enhance the self-management of pain, with improved outcomes reflective of the biopsychosocial model of pain targeting the three self-management tasks. These tasks include medical (e.g., taking medications, exercising), role (e.g., changing behaviors, such as becoming more active instead of sedentary), and emotional management (e.g., learning to deal with anxiety or a fear of pain) [14]. The medical, role, and emotional management tasks were reflected in our study findings through the enhanced self-management of pain, improved pain outcomes with decreased pain intensity, less anxiety, and improved sleep. We obtained supporting evidence for our theory-based intervention according to the qualitative insights from our participants. Self-management is also described by five core skills, namely problem solving, decision-making, utilizing resources, forming partnerships with healthcare providers, and taking action [14]. Patients who self-manage adequately are able to clearly define their challenges, devise possible solutions, implement these, and then evaluate the results. They make logical, informed decisions (e.g., knowing when they need to rest and resume exercise based on their pain level). They seek pain resources to educate themselves about their pain, collaborate with their healthcare providers (e.g., discussing the appropriate use of pain medications), and form realistic action plans (e.g., walking for 30 min at least three times a week). These are important skills with strong implications for patients and healthcare providers. Our findings demonstrate examples of how these core self-management skills were portrayed by the participants, especially as their pain levels improved with the intervention. Participants addressed their pain actively using APA, made decisions, and took action (e.g., participating in a walk and pacing to finish) in order to continue to enjoy their lives while overcoming their fear of pain, as well as reviewing other APA resources and consulting with their healthcare providers.

Our findings also indicate that the delivery of our intervention via technology was successful and the APA use was sustained by participants for up to 3 months of self-administration, which contributed to participants’ ability to self-manage their pain, with some reduction in healthcare utilization and analgesic use. Overall, participants described their experiences by evaluating the impact of APA on their pain and pain-related outcomes. They also described their experiences related to how APA worked for them (feasibility and sustainability) and how they experienced our intervention components, including those that they did not find very helpful, and provided recommendations. Altogether, the participant feedback provided support to our theory-driven APA intervention to self-manage pain.

Self-management alone may not be sufficient for effective, sustained pain management [49]. Instead, incorporating self-management with an active intervention (e.g., APA) could be key. For example, providing a theory-driven and self-administered, hands-on intervention utilizing APA could facilitate the continued self-management of pain; this could be a very useful, innovative pain modality. In addition, we leveraged a digital healthcare approach in the delivery of our intervention. Digital healthcare can facilitate increased access to minimize pain care disparities [44]. Digital healthcare interventions are transforming healthcare and play a pivotal role in individualizing care by using a patient-centered approach, while simultaneously providing the ability to intervene at a larger scale with a wider, global reach. Other studies have investigated auriculotherapy (the stimulation of ear points) for chronic pain [50,51,52,53,54,55]. Studies specific to APA leveraging technology are lacking; hence, there is value in implementing and testing theory-based interventions to facilitate well-designed trials in larger studies. Remotely delivered APA can be pivotal in providing an accessible pain modality that is valuable to patients and healthcare providers.

While no single theory is sufficient to guide intervention development, we nevertheless found that our theory-driven intervention facilitated the self-management of pain. Overall, our intervention allowed participants to assume an active role in self-managing their pain. However, the nature of this study prevented us from identifying the conditions resulting in participants’ chronic pain and exploring the frequency of certain experiences (e.g., pain flare-ups). Other study limitations included potential bias and challenges in relation to qualitative descriptive research, thematic analysis, and the adequacy of the sample sizes, but these approaches were chosen as they were most appropriate to the study, details were described to enhance the rigor, and data saturation was achieved.

Some future recommendations based on our works include emphasizing the motivational messages and self-monitoring of data to facilitate self-efficacy toward solidifying the self-management of pain; these need to be enhanced in future studies. Using a web-enabled app to increase self-learning and access, as well as disseminating video vignettes of patients’ successful use of APA on our study website, may also increase its adherence and sustainability. Further, larger samples and investigation of the intervention’s efficacy in a pragmatic clinical trial will be important to advance the evidence for our pain management intervention.

## 5. Conclusions

There is a limited number of research studies that specify or detail interventions that are founded on or guided by a theoretical framework. We adapted Bandura’s theory based on his proposed four major self-efficacy sources (personal accomplishment, vicarious experience, verbal persuasion, emotional arousal) to guide the development of our intervention. Since self-efficacy was intended to impact the self-management of chronic pain and improve pain outcomes, the main outcomes assessed in our studies targeted the key self-management tasks (medical management, role management, emotional management). In applying and evaluating Bandura’s self-efficacy theory using APA delivered via digital healthcare technology, we were able to optimize our intervention toward improving pain outcomes, including APA adherence, sustainability, and retention. Further research is needed to refine our adapted model and advance its value in managing chronic pain. Nevertheless, the work presented here can be used to guide the development, implementation, and evaluation of health behavior change interventions for chronic pain and other disorders.

## Data Availability

The data that support the findings of this study are available from the corresponding author (J.K.) upon reasonable request.
